# The Influence of Contextual Predictability on Word Segmentation in Chinese Reading: An Eye-Tracking Study

**DOI:** 10.3390/bs16020185

**Published:** 2026-01-27

**Authors:** Mengchuan Song, Wenxin Zhang, Yashu Cao, Jingxin Wang

**Affiliations:** 1 Faculty of Psychology, Tianjin Normal University, Tianjin 300387, China; 2 Tianjin Social Science Laboratory of Students’ Mental Development and Learning, Tianjin 300387, China; 3 Key Research Base of Humanities and Social Sciences of the Ministry of Education, Academy of Psychology and Behavior, Tianjin Normal University, Tianjin 300387, China

**Keywords:** word segmentation, contextual predictability, overlapping ambiguous strings

## Abstract

Word segmentation is a fundamental component of lexical processing, and Chinese reading—lacking inter-word spacing—requires readers to identify word boundaries based on prior experience. Previous studies have shown that contextual predictability facilitates lexical identification in Chinese reading; however, its influence on word segmentation remains unclear. This study used eye-tracking to examine the relationship between contextual predictability and readers’ segmentation preferences during Chinese sentence reading. Overlapping ambiguous three-character strings (e.g., 花生长) were used as the region of interest (ROI), and a 2 (segmentation type: AB-C (e.g., 花生/长) vs. A-BC (e.g., 花/生长)) × 2 (contextual predictability: high vs. low) within-subjects design was adopted. A total of 76 native Chinese speakers completed the task. The results showed that contextual predictability had a significant effect on skipping probability: Highly predictable target character strings were skipped more often than low-predictability words. However, contextual predictability did not influence any eye-movement measure. In contrast, segmentation type produced consistent effects across all measures, with shorter reading times for AB-C than for A-BC, indicating a stable preference for two-character segmentation. More importantly, no interaction emerged between contextual predictability and segmentation type, and Bayesian model comparison further supported this conclusion. These findings suggest that Chinese reading involves a robust preference for AB-C segmentation and that contextual predictability and word segmentation operate as independent processes, with predictability exerting minimal influence on word segmentation during reading. This result supports the Chinese Reading Model (CRM).

## 1. Introduction

The word serves as the fundamental unit of reading processing. Word segmentation refers to the process of delineating and identifying word boundaries within sentences or passages during reading activities. This process occupies a central position in the cognitive mechanisms of information processing and constitutes the primary stage of lexical processing (Zheng, 1981).

Compared with alphabetic writing systems, the Chinese writing system exhibits distinct structural characteristics. In alphabetic languages, spaces between words visually demarcate word boundaries, allowing readers to rely on explicit spatial cues for rapid word recognition and semantic integration. The inter-word spacing thus serves as a critical factor in segmenting words during reading. In contrast, Chinese script is presented as a continuous sequence of characters without visible spaces or other physical markers to separate words. Because individual Chinese characters can independently convey meaning or combine with others to form multi-character words, word boundaries are not visually salient and must instead be inferred through readers’ prior experience and top-down cognitive mechanisms. This feature distinguishes Chinese reading from that of alphabetic scripts in terms of cognitive processing: word segmentation in Chinese not only constitutes a prerequisite for lexical identification but also underlies syntactic parsing and global semantic comprehension. Failure to accurately identify word boundaries during reading can directly hinder sentence-level understanding. Therefore, investigating how Chinese readers achieve word segmentation during reading is crucial for understanding sentence processing in the Chinese language.

Previous studies have primarily employed inter-word spacing as an aid to investigate segmentation processes in Chinese reading (Hsu & Huang, 2000; Bai et al., 2008; Shen et al., 2010; Blythe et al., 2012; N. N. Zhou & Jie, 2018). Some researchers argued that when readers encounter ambiguous or complex texts, the insertion of spaces can facilitate text comprehension effectively (Hsu & Huang, 2000; Shen et al., 2010; Blythe et al., 2012). In their studies, readers were found to improve reading efficiency and enhance sentence comprehension accuracy, particularly when processing novel or unfamiliar words, by relying on the visual cues provided by inter-word spacing. However, other studies reported that inter-word spacing does not exert a significant influence on Chinese readers’ reading efficiency (Bai et al., 2008; N. N. Zhou & Jie, 2018). These researchers suggest that readers primarily depend on their prior linguistic knowledge rather than on spacing cues to segment words. For example, some researchers argue that Readers make use of the semantic context during reading to assist with word segmentation and text comprehension (de Almeida & Libben, 2005; Pollatsek et al., 2010; Huang & Li, 2020).

Contextual predictability refers to the probability that an upcoming word can be anticipated by readers based on the preceding linguistic context (Rayner & Well, 1996). This measure reflects the impact of contextual information on word recognition and sentence comprehension. A substantial body of research has demonstrated that contextual predictability significantly affects lexical processing in both alphabetic and Chinese reading. In alphabetic languages, highly predictable words are associated with increased skipping rates, reduced fixation durations, and faster sentence processing (Rayner & Well, 1996; Rayner et al., 2001, 2005; Kliegl et al., 2004; Staub, 2015; Willems et al., 2016). In studies of Chinese reading, Rayner et al. (2005) found that words with low contextual predictability are more likely to be fixated and receive longer fixation times from readers. In addition, Zhao et al. (2019) used eye-tracking technology to find that words with high contextual predictability have higher skipping rates, shorter reading times, and fewer regressions, indicating that contextual predictability facilitates word recognition. Liu et al. (2020) found that in Chinese reading, the effects of contextual predictability and factors such as word frequency are independent of each other. Contextual predictability can promote readers’ pre-visual processing of target words, thereby increasing the probability of skipping those words.

In studies investigating the relationship between contextual predictability and word segmentation in alphabetic languages, overlapping ambiguous words are typically used as experimental materials (de Almeida & Libben, 2005; Pollatsek et al., 2010). Overlapping ambiguous words typically exhibit two possible interpretations. For example, the English word *unlockable* consists of three morphemic components: *un-lock-able*. When the prefix (*un-*) combines with the root (*lock*), it forms a left-branching structure (*unlock-able*). Conversely, when the suffix (*-able*) attaches to the root, it yields a right-branching structure (*un-lockable*). de Almeida and Libben (2005) found that the interpretation of such ambiguous words is influenced by the sentence context in which they appear. In sentences with weak contextual constraints, ambiguous words tend to show a left-branching segmentation advantage, whereas in strongly constraining contexts, readers are more likely to adjust their segmentation preference according to the overall semantic meaning of the sentence. Similarly, Pollatsek et al. (2010) embedded overlapping ambiguous words in different sentence contexts and manipulated contextual constraint to examine readers’ segmentation preferences and whether these preferences were modulated by context. The results revealed a left-branching bias among readers, which was likely due to the higher frequency of *un-X* formations compared to *X-able* formations. The result is consistent with their findings in previous studies, demonstrating that the frequency of the components constituting a word has a stable effect on the fixation time of longer compound words (Hyönä & Pollatsek, 1998; Niswander-Klement & Pollatsek, 2006). Moreover, this study also found that when the context favored the right segmentation, ambiguous words received more ambiguous fixations than those presented in neutral contexts. In contrast, when favoring the left segmentation, readers showed no clear preference for the ambiguous words. In Chinese reading, Huang and Li (2020) examined readers’ word segmentation using overlapping ambiguous strings as target items. Ambiguous strings (three-character strings; ABC), in which the middle character can create distinctive words with the characters to both its left (word AB) and its right (word BC; Gan et al., 1996). For example, in the string 领队长, the string cannot only be segmented as 领队/长, but also 领/队长. Unlike overlapping ambiguous words in English, such strings in Chinese do not possess legitimate morphological structures; their actual interpretations depend on readers’ dynamic segmentation during reading. The results showed that readers exhibited a clear preference for the left-branching segmentation AB-C during processing.

Word segmentation is achieved through a top-down processing mechanism during reading. Specifically, readers can utilize contextual information to predict upcoming words during the preview stage, thereby facilitating segmentation. Based on this perspective, a series of theories and models have been proposed to explain the mechanisms underlying word segmentation. Hoosain (1991) argued that lexical recognition and word segmentation constitute an inseparable unified process: only after individual Chinese characters are recognized can readers determine word boundaries by integrating bottom-up visual information with top-down semantic information. The hybrid representation model posits that the mental lexicon contains both morphemic and whole-word representations, and the recognition of compound words results from the interaction of these two levels (Pollatsek et al., 2010; Libben et al., 2020). During word recognition, both morphemic and whole-word representations are activated and interact to achieve segmentation. Perfetti and Tan (1999) proposed the dual-character assembly strategy, which assumes that the first two characters of overlapping ambiguous strings hold absolute priority in forming words. Readers tend to treat these initial two characters as a single word, and when this tendency conflicts with the contextual information, it leads to longer word segmentation times.

On the other hand, Li et al. (2009) developed a computational model of Chinese word recognition and segmentation by adapting the interactive activation model (McClelland & Rumelhart, 1981). The model posits that word recognition and segmentation constitute a unified, interdependent process: segmentation occurs only after a word has been recognized. Within the visual field, character processing operates in parallel; however, characters located farther from the visual focus are recognized more slowly. Furthermore, the word recognition process itself is serial: once a word is successfully identified, its constituent character units are inhibited, thereby allowing subsequent word recognition processes to proceed more efficiently.

Traditionally, because Chinese, unlike alphabetic scripts, lacks overt word boundaries, some studies have proposed that the basic unit of Chinese reading is the character rather than the word (Chen, 1992; H. M. Yang & McConkie, 1999; Chen et al., 2003; Tsai & McConkie, 2003). For example, the decomposed storage view argues that two-character words are represented and processed at the character level (B. Zhang & Peng, 1992). However, recent studies have found that Chinese readers tend to process longer words as holistic units, even when some of their constituent characters can independently form other words (J. Yang et al., 2012; J. Zhou & Li, 2021). Li and Pollatsek (2020) proposed the CRM, which suggests that characters within the perceptual span are processed in parallel, and all words composed of these characters are activated. In this competitive process, two-character words (AB) receive excitatory feedforward connections from both constituent character units (A and B), giving them a processing advantage over single-character words (A). Similarly, Liu et al. (2020) also found that readers make use of contextual predictability to facilitate the parafoveal processing of two-character target words, thereby influencing the saccade amplitude directed toward those words. Huang and Li (2020) used overlapping ambiguous strings as ROIs to examine the effects of contextual predictability. Their results showed that although readers do use contextual information to aid word segmentation during reading, such use is not fully sufficient.

In summary, during Chinese reading, readers tend to process two-character words as unified lexical units rather than analyzing them character by character in sequence. Most existing studies on the influence of contextual predictability on Chinese lexical processing have examined two-character words as target items. However, Chinese reading involves a large number of overlapping ambiguous strings. Tsang et al. (2025) found that about 85% of the experimental sentences in their study contained at least one ambiguous word boundary, and participants disagreed on the position of word boundaries in 8.96% of the cases. Although a few studies have investigated the segmentation of such ambiguous items (Huang & Li, 2020), within these studies each sentence frame typically allows only one segmentation to yield a coherent meaning, while the alternative segmentation produces a semantically anomalous sentence. This limitation raises the possibility that readers’ segmentation preferences may stem from the requirement for semantic coherence rather than from contextual predictability itself. Therefore, how readers use contextual information to guide word segmentation during reading remains an issue in need of further investigation. Accordingly, the present study aims to examine how contextual predictability operates in Chinese word segmentation and, in particular, whether context can modulate readers’ segmentation preferences when processing overlapping ambiguous strings.

The present study employed overlapping ambiguous strings as experimental materials. For example, in the word 花生长 (hua-sheng-zhang), the character 生 can combine with the preceding character 花 to form 花生 (“peanut”) or with the following character to form 生长 (“grow”), resulting in two possible segmentations. Using this word as the target, we constructed experimental sentences such as 屋后的花生长得很旺盛, in which both segmentations yield semantically plausible sentences, making it impossible to disambiguate based solely on the sentence context. Thus, the influence of semantic information can be ruled out, allowing for an isolated examination of how contextual information affects word segmentation. Furthermore, because Chinese lacks explicit word boundary cues such as spaces that provide word segmentation information in alphabetic languages, readers must rely on contextual information to process sentences. Contextual predictability thus constitutes an important factor influencing Chinese reading. We also examine the role of contextual predictability in Chinese reading.

Accordingly, the present study investigates the effects of contextual predictability and Chinese word segmentation on Chinese reading, as well as whether an interaction exists between the two. The present study proposes the following hypotheses:

(a) Contextual predictability facilitates readers’ processing, such that reading efficiency is higher under conditions of high predictability.
(b) In Chinese reading, readers exhibit a stable tendency to segment words into two-character units; readers need less time when processing the string AB-C.
(c) If contextual predictability directly influences readers’ segmentation preferences, an interaction between the two factors should emerge; otherwise, their effects on Chinese reading can be considered relatively independent.

## 2. Materials and Methods

### 2.1. Participants

The calculations of G*Power 3.1 were conducted for the four-level within-subjects experimental design applied in this study. The total sample size predicted a level of statistical power of 0.8, the effect size was set at f = 0.25, the significance level (α) at 0.05 required at least 24 participants. Although the a priori power analysis suggested that a minimum of 28 participants would be sufficient under ideal conditions, the experimental data were inevitably influenced by multiple sources of variability, for example, individual differences in reading ability. To minimize the influence of such factors and reducing sampling-related error introduced by participant selection, we recruited a larger sample of 76 participants for the formal experiment to improve the ecological validity and robustness of the findings. In this experiment, participants were 76 university students aged 18–25 (*M* = 20.5). All participants were native Mandarin speakers who were naive to the purpose of the study and provided written informed consent prior to participation.

### 2.2. Stimuli and Design

This study employed a 2 (segmentation type: AB-C vs. A-BC) × 2 (contextual predictability: high vs. low) within-subjects experimental design. The experimental materials consisted of sentences containing 32 pairs of overlapping ambiguous three-character strings (ABC) as target character strings (e.g., “花生长” hua-sheng-zhang). Each sentence frame included four experimental conditions: (a) AB-C/high predictability, (b) AB-C/low predictability, (c) A-BC/high predictability, and (d) A-BC/low predictability. Each participant was required to read a total of 128 sentences in the formal experiment. The sentence length ranged from 21 to 32 characters. To minimize potential confounding factors, the first (A) and last (C) characters of each target character string did not form lexical units with adjacent characters, and the target character strings appeared at least two characters away from the end of the sentence. Subsequently, a group of university students who did not participate in the formal experiment were invited to evaluate the experimental materials.

Independent-sample *t*-tests were conducted to examine differences in word frequency and stroke count between the AB and BC segments, and the results showed no significant differences (word frequency: *t* = 0.33, *p* = 0.74; stroke count: *t* = 1.42, *p* = 0.16). Subsequently, the overlapping ambiguous strings (ABC) were presented individually to 32 undergraduate participants, who were asked to segment them. During this process, readers may either accept both segmentation alternatives or regard only one of them as plausible. For example, for the string “花生长”, readers may consider both “花生/长” and “花/生长” as acceptable segmentation outcomes, or they may judge only “花/生长” as the sole valid segmentation. The results indicated the preference of participants for segmenting the sentences, which refers to participants’ preference for a particular word segmentation pattern, that is, their tendency to adopt a specific segmentation in the absence of contextual constraints. The results showed that the difference between the two segmentation patterns was not significant (*t* = 0.13, *p* = 0.89) (see Table 1).

The complete sentences were presented without indicating the correct segmentation, and 32 university students were asked to segment the overlapping ambiguous strings (ABC). Their accuracy rates were recorded, and sentences in which the direction of contextual predictability was consistent with the intended segmentation and that showed better control were selected to enhance the reliability of the contextual predictability manipulation. This measure reflects the likelihood that the string is correctly segmented by participants under a given contextual condition. In the final set of experimental sentences, the accuracy of word segmentation did not differ significantly across the four experimental conditions (*F* = 1.63, *p* = 0.19), and there was a high degree of predictability between the contextual information and the correct segmentation.

Because the present study used ambiguous strings as target items, readers were unable to predict the target using a traditional cloze task. Another group of 32 undergraduate students was recruited to evaluate the contextual predictability of the sentences. They were asked to judge the extent to which the preceding context predicted the segmentation of the target character string (AB-C or A-BC). The results revealed a significant difference between high- and low-predictability sentences, *t*(31) = 19.28, *p* < 0.001. Subsequently, 24 undergraduate students were asked to rate the predictability of the target word (“AB” or “A”) in the following clause based on the preceding context (from the beginning of the sentence to the comma) using a 7-point rating scale. In addition, no significant differences in sentence fluency were observed among the four experimental conditions—*F*(1, 31) = 0.41, *p* = 0.74 (see Table 2)—which indicates that all experimental sentences were fluent.

The college students who participated in the material ratings did not take part in the formal experiment. Examples of the materials are shown in Table 3.

### 2.3. Apparatus and Procedure

Stimuli were presented on a high-resolution 24-inch Benq LCD monitor (1920 × 1080 pixels) with a fast refresh rate (144 Hz). The experimental materials were displayed in Song 32-point font as black text on a white background. At 60 cm viewing distance, each character subtended about 1°. An Eyelink 1000 Plus eye (SR Research, Kanata, ON, Canada) tracker recorded right-eye movements during binocular reading at a sampling rate of 1000 Hz.

Before all, Participants were presented with the instruction text and the experiment was explained to them. A three-point calibration was then performed for each participant. Practice trials were then displayed to ensure that participants fully understood the task requirements. In the formal experiment, participants were randomly assigned to one of four sets of experimental materials. Before each sentence, a fixation cross (“+”) was displayed, and participants were instructed to fixate on it until it disappeared, after which the sentence appeared. Upon finishing reading each sentence, participants pressed the space bar to proceed to the next trial. For half of the sentences, a comprehension question related to the main idea of the sentence followed, requiring participants to make a “yes” or “no” judgment based on the sentence content. The entire experiment lasted approximately 12 min.

## 3. Results

**Figure 1:**
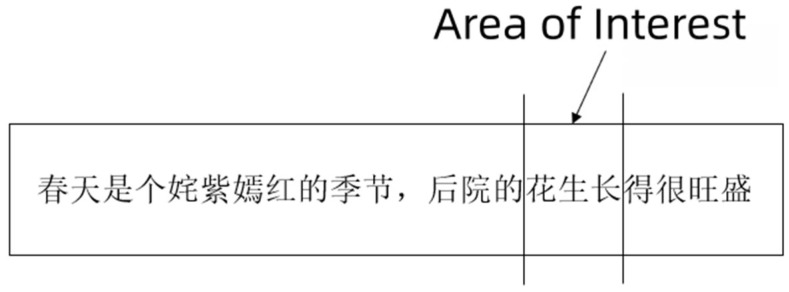
An example of AOI. (The sentence translate as: Spring is a season full of vibrant colors, and the flowers (segment in 花-生长)/peanuts (segment in 花生-长) in the garden grow vigorously.)

In this study, the selection of AOI was consistent with previous studies, in which the overlapping ambiguous string (ABC) was treated as a single unit for analysis (Ma et al., 2014; Huang & Li, 2020; Huang et al., 2021), in order to comprehensively evaluate how readers process the entire overlapping ambiguous string. An example of AOI is shown in Figure 1. The possible meanings of the sentence are:

A-BC: Spring is a season full of vibrant colors, and the flowers in the garden grow vigorously.

AB-C: Spring is a season full of vibrant colors, and the peanuts in the garden grow vigorously.

The lme4 package (Bates et al., 2015) in R 4.5.1 (R Development Core Team, 2025) was used to analysis the data. Continuous variables were fitted using linear mixed-effects models, whereas binary variables were fitted using generalized linear models. In the statistical analyses, segmentation type (AB-C vs. A-BC), contextual predictability (high vs. low), and their interaction were specified as fixed factors to examine their systematic effects on reading processes. To account for unsystematic variability arising from individual differences and item-related differences, participants and items were included in the models as random factors.

Following previous studies (de Almeida & Libben, 2005; Huang & Li, 2020; Huang et al., 2021; Ma et al., 2014; Pollatsek et al., 2010), the present research examined several eye-movement measures on the target character strings, including skipping rate, first fixation duration (FFD), single fixation duration (SFD), gaze duration (GD), total reading time (TRT), selective regression path reading time (SRP), and second-pass reading time (SPRT).

Before analysis, data were first removed according to the following criteria: (a) fixations shorter than 80 ms or longer than 1000 ms; (b) trials in which head movements or other disruptions occurred during the experiment (2 trials in total); (c) fewer than five fixations occurred within a sentence (28 trials in total). In sum, 30 trials were removed.

### 3.1. Frequentist Statistical Tests

Contextual predictability exhibited a significant main effect on skipping rate (|*z*| = 2.04, *p* = 0.04); participants showed a higher skip rate under the high-context predictability condition. No significant differences were observed for FFD (*t* = 1.57, *p* = 0.12); SFD (*t* = 0.84, *p* = 0.41); GD (|*t*| = 0.28, *p* = 0.78); TRT (*t* = 0.76, *p* = 0.45); SRP (*t* = 0.01, *p* = 0.99) and SPRT (*t* = 0.79, *p* = 0.43).

In contrast, the main effect of segmentation type was not significant for skipping rate (*z* = 1.40, *p* = 0.16), yet significant differences were found across FFD (*t* = 3.12, *p* < 0.01); SFD (*t* = 2.94, *p* < 0.01); GD (*t* = 2.06, *p* = 0.04); TRT (*t* = 2.54, *p* < 0.01); SRP (*t* = 2.54, *p* = 0.01) and SPRT (|*t*| = 3.32, *p* < 0.01). Compared to the AB-C pattern, participants needed more time for the A-BC word segmentation.

Moreover, the interaction between contextual predictability and segmentation type did not reach significance for skipping rate (*z* = 0.18, *p* = 0.86) or any of the eye-movement measures (FFD (*t* = 0.06, *p* = 0.95); SFD (*t* = 1.13, *p* = 0.26); GD (*t* = 0.01, *p* = 0.99); TRT (|*t*| = 0.39, *p* = 0.70); SRP (|*t*| = 0.39, *p* = 0.70) and SPRT (|*t*| = 0.54, *p* = 0.59)).

**Figure 2:**
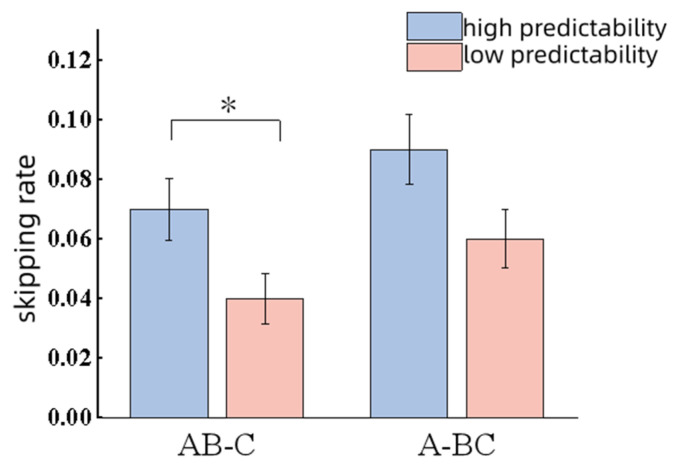
Results of skipping rate. Note. * *p* < 0.05.

**Figure 3:**
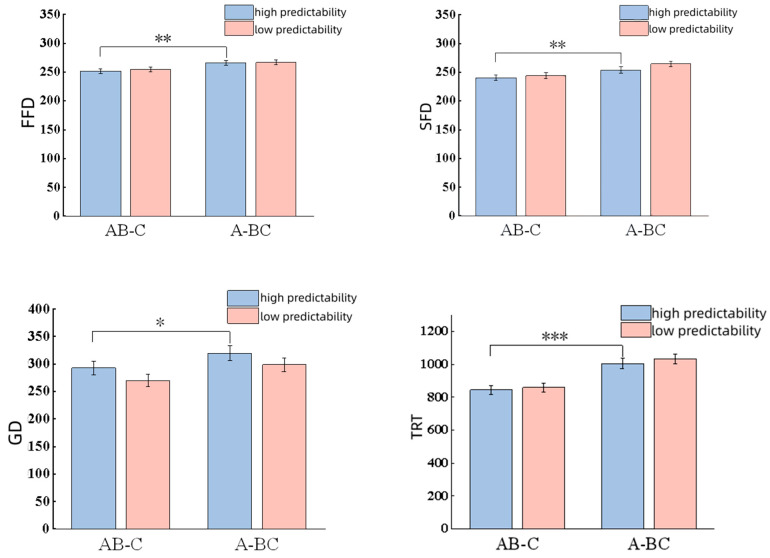

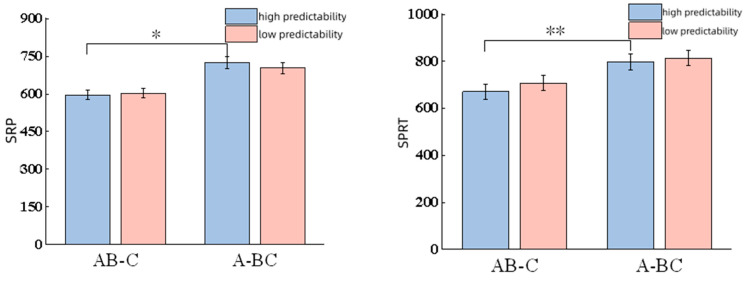
Results of time measures. Note. * *p* < 0.05, ** *p* < 0.01, *** *p* < 0.001.

All data are shown in Figure 2 and Figure 3.

### 3.2. Bayesian Analysis

We further examined the model using Bayesian analysis to improve the accuracy of data analysis. Specifically, the full model included fixed effects for the main effects of contextual predictability, segmentation type, and their interaction. For the model aimed at testing the interaction, only the main effects of contextual predictability and segmentation type were included as fixed factors. Bayesian analysis revealed that, in the target character strings (ABC) region, there was no significant interaction between contextual predictability and segmentation type across all measures (FFD: *BF* = 0.24; SFD: *BF* = 0.24; GD: *BF* = 0.24; SRP: *BF* = 0.22; TRT: *BF* = 0.17; SPRT: *BF* = 0.19). When the Bayes factor is smaller than 1, it indicates that the data provide greater support for the null hypothesis than for the alternative hypothesis. In this study, all BF values ranged from 0.17 to 0.24, providing moderate evidence in favor of the absence of an interaction between contextual predictability and segmentation type.

## 4. Discussion

This study employed overlapping ambiguous words as target stimuli to examine the effects of contextual predictability and segmentation type on Chinese reading. Consistent with previous findings, a stable effect of contextual predictability was observed in the skipping rate: compared to the low-predictability condition, participants exhibited more skipping under the high-predictability condition, indicating that readers were more likely to skip the target word when its predictability was high. Although the main effect of segmentation type on skipping rate was not significant, it showed robust effects across six temporal measures. Specifically, reading times for the overlapping ambiguous string ABC were shorter in the AB-C condition than in the A-BC condition, suggesting that readers tended to segment text into two-character words—a finding that supports the assumptions of the CRM. No significant interaction was found between contextual predictability and segmentation type across all measures, indicating that contextual predictability does not modulate the word segmentation process in Chinese reading.

Consistent with previous findings, a robust contextual predictability effect was observed in the skipping rate (Rayner et al., 2005; Kliegl et al., 2010; Schotter et al., 2012; Liu et al., 2020). As an indicator reflecting the proportion of words skipped during reading, the skipping rate directly captures the influence of contextual predictability on reading behavior. The significantly higher skipping rate under the high-predictability condition suggests that readers can effectively utilize contextual information to anticipate upcoming words, thereby reducing processing demands and enhancing overall reading efficiency (M. M. Zhang et al., 2020). Rayner et al. (2005) and Schotter et al. (2012) found that sentences with high contextual predictability provide richer contextual cues, thereby facilitating readers’ parafoveal preview and lexical processing of target words, increasing both the likelihood and amplitude of saccades. In the present study, we also observed no significant differences between high- and low-predictability conditions in FFD, SFD, or GD.

However, other studies have reported that contextual predictability significantly affects temporal measures (Rayner et al., 2001, 2004, 2005, 2011; Staub, 2015). In these studies, words in low-predictability contexts were more likely to be fixated and to receive longer fixation durations, whereas high contextual predictability led to shorter fixation times and faster lexical processing. The inconsistency of these results suggests that contextual predictability may exert a relatively independent influence during lexical processing. In the present study, contextual predictability may primarily modulate early anticipatory or integrative processes, such as pre-activation of upcoming lexical candidates and sentence-level semantic integration, rather than the duration of fixation once a word has already been fixated. Specifically, its effects on word recognition and integration may operate independently of other variables, such as word frequency or parafoveal preview. In other words, contextual predictability can activate top-down semantic integration through syntactic and contextual cues, thereby reducing the cognitive load of word recognition and sentence comprehension. This pathway is distinct from the bottom-up passive input driven by factors such as word frequency and orthographic form and may instead represent an active top-down process.

In addition, the results revealed a stable tendency toward disyllabic word segmentation, indicating that readers preferred to segment overlapping ambiguous strings as disyllabic words (AB-C) rather than monosyllabic words (A-BC). It should be clarified that, unlike English studies that use ambiguous words as target items (de Almeida & Libben, 2005; Pollatsek et al., 2010), the present study examined sentences constructed with ambiguous strings. Unlike overlapping ambiguous words, which have legitimate morphological structures in the mental lexicon, such strings lack stable lexical representations, and their segmentation and interpretation rely on readers’ dynamic inferences during the reading process. The stable tendency may be attributed to the fact that, in Chinese reading, the word serves as one of the fundamental units of language, possessing relatively independent meaning and function. Compared with individual characters, words exhibit greater stability in terms of semantics, syntax, and frequency. Thus, when a word is processed as an integrated unit, the constituent characters are constrained by the word’s overall semantic and structural properties, facilitating recognition and comprehension. Moreover, this pattern may also stem from the dominance of disyllabic words in modern Chinese (more than 70%), which makes readers more likely to process the first two characters of overlapping ambiguous strings as a disyllabic word.

This result supports the view of the Chinese Reading Model (CRM), which posits that disyllabic words are more easily processed during Chinese reading. According to this model, during each fixation, the characters within the reader’s perceptual span are activated. For example, when the reader’s fixation point falls on the first character “花” of the word “花生” (“peanut”), the perceptual span may simultaneously cover “花”, “生” and subsequent characters. Once these characters are visually activated, the activation is transmitted forward through feed-forward connections to both the character and lexical levels. At the lexical level, all possible candidate words (including disyllabic words) are activated. If additional characters fall within the perceptual span (for instance, “长” follows after “花生”), potential disyllabic words “花生” and “生长” will both be activated and compete at the lexical level. During this competition process, the word with the highest level of activation ultimately prevails and is segmented from the text. Once “花生” is successfully recognized, its constituent characters “花” and “生” are marked as processed. Then the subsequent processing will skip these characters.

During reading, when readers encounter the word “花生”, both the disyllabic word “花生” and the monosyllabic word “花” are activated. However, “花生” receives a higher level of activation as a disyllabic word. When the reader fixates on “花”, only one character unit provides excitatory feed-forward input, whereas processing “花生” allows for excitatory feed-forward activation from two-character units. In other words, compared with monosyllabic words, disyllabic words can obtain more support from the textual context during reading. This competitive mechanism increases the efficiency of word recognition, leading to a word superiority effect in which disyllabic words are more easily segmented from the sentence than monosyllabic words.

However, the interaction between contextual predictability and word segmentation did not reach significance across any measure under the present manipulation. Contextual predictability does not exert a modulatory effect on the word segmentation process during sentence reading, indicating that the two processes operate independently. This pattern differs from the findings reported by Huang and Li (2020), who observed a significant interaction between contextual predictability and segmentation in early eye-movement measures. One plausible explanation for the discrepancy concerns the nature of the contextual manipulation. In their study, the contrast between highly predictable contexts and neutral contexts created a substantial difference in contextual support, such that only the highly constraining contexts enabled readers to strongly anticipate a specific segmentation of the ambiguous string. In contrast, the present study manipulated high versus low predictability, and the two levels were both constraining to some extent. Under these conditions, both segmentations of the ambiguous string remained reasonably predictable, and even items in the low-predictability condition could still be anticipated to some degree. Therefore, the interaction did not reach statistical significance. In future research, it will be necessary to further control the levels of contextual predictability in order to determine how readers segment ambiguous strings and process sentences under different degrees of contextual constraint.

This finding supports the graded pre-activation perspective, which posits that contextual predictability progressively activates the semantic and syntactic information of words rather than directly intervening in the early word segmentation process (Staub, 2015). Such activation is based on the probability of word occurrence, with highly predictable words exhibiting shorter fixation durations and higher skipping rates during processing (Su et al., 2016). The main effect of contextual predictability in the present study was primarily reflected in skipping rates. Contextual information incrementally enhances the activation of candidate lexical items in a probabilistic manner, yet such enhancement emerges mainly when lexical identification is approaching the stage of semantic integration—for instance, in the form of higher skipping rates for highly predictable words—rather than directly contributing to the establishment of visual word boundaries. Bayesian analyses further suggest that the influence of context likely occurs at the stages of lexical activation and semantic integration. Contextual predictability thus appears to exert its effects gradually throughout the processing stream, rather than directly determining the formation of lexical boundaries.

Moreover, the absence of this interaction under the present experimental conditions further indicates that, in Chinese reading, contextual predictability may primarily enhance reading efficiency by facilitating word recognition and sentence comprehension, rather than directly influencing the word segmentation process. Word segmentation predominantly occurs during the word recognition stage, whereas the effects of contextual predictability are largely manifested during the semantic integration stage. Previous research has shown that, in Chinese reading, the structural properties of words—such as word frequency, word length, and morphemic information—play a dominant role in the word segmentation process. For example, high-frequency words, due to their greater occurrence in the language, are more familiar to readers in terms of their structural characteristics and are therefore more easily recognized and segmented (Li et al., 2009; Ma et al., 2014). During the word segmentation stage, readers are required to rapidly recognize words and segment the sentence, whereas the integration of contextual information demands more time and cognitive resources. Consequently, the influence of contextual information primarily occurs during subsequent semantic processing, whereas its role during the word segmentation stage is relatively limited. In other words, readers may rely more heavily on other sources of information—such as word frequency—to facilitate the completion of the word segmentation process (Ma et al., 2014; Huang & Li, 2020, 2024).

The CRM proposes that word segmentation and lexical identification are not two serially ordered stages but a unified process occurring simultaneously. During reading, readers integrate parafoveal information from multiple positions within the perceptual span and activate potential candidate words in parallel. Word segmentation is accomplished through competition among all candidate words, whereby the winning candidate is selected and identified. This process relies primarily on the internal structural properties of the lexical string and the activation levels of neighboring words, rather than on the direct modulation of contextual predictability. Contextual predictability exerts a stronger influence on the pre-activation of upcoming words, rather than directly entering the early-stage competition involved in word segmentation. In the present study, no significant interaction between contextual predictability and segmentation type was observed across multiple eye-movement measures, and Bayes factor values (BF < 0.3) further supported the absence of such interaction, indicating that context did not alter the processing differences associated with distinct segmentation patterns. This result is consistent with the predictions of the CRM, in which contextual information may influence overall reading fluency and skipping tendencies to some extent, with its effects manifested primarily at the level of lexical activation. In the present study, readers exhibited a stronger preference for bisyllabic word segmentation (AB-C) during the word segmentation process. Even when contextual information was available, readers’ preference for segmentation patterns driven by string-internal features was not attenuated.

Importantly, the present study also found that contextual predictability yielded a significant main effect only on skipping rates, with no significant effects on other eye-movement measures. This pattern further suggests that context primarily facilitates higher-level reading strategies, such as skipping highly predictable words, while exerting limited influence on lower-level segmentation processes and reading performance. In contrast, segmentation type produced robust differences in timing measures, revealing a strong tendency toward two-character segmentation (AB-C), indicating that the structural properties of the string have a stable and substantial impact on Chinese reading—an impact not altered by variations in contextual predictability. Taken together, these findings are consistent with the theoretical framework of the CRM and provide new insights into Chinese reading under naturalistic conditions: Chinese word segmentation is highly dependent on character-level features and parafoveal processing, and future research should further examine the contextual or cueing conditions under which segmentation tendencies and reading performance may be modulated.

## 5. Conclusions

This study employed eye-tracking technology to investigate the influence of contextual predictability on word segmentation in Chinese reading. The results showed that contextual predictability facilitated readers’ processing performance, with higher skipping rates observed in highly predictable contexts. There is a stable tendency for two-character word segmentation (AB-C) in Chinese reading, and contextual predictability and word segmentation operate as independent processes, with contextual predictability exerting minimal influence on segmentation during reading. These findings support the graded pre-activation perspective and the Chinese Reading Model (CRM).

## Figures and Tables

**Table 1:** Target-word-related statistics *M* (*SD*).

	AB (“花生”)	BC (“生长”)	*t*	*p*
stroke count	15.13 (4.59)	16.81 (4.88)	1.42	0.16
word frequency	242.25 (725.94)	194.13 (376.41)	0.33	0.74
segmentation pattern preference	20.84 (9.37)	21.16 (9.37)	0.13	0.89

Note. Means and standard deviations (SD), where SD are provided in brackets ().

**Table 2:** Material rating information *M* (*SD*).

	High-Predictability Words		Low-Predictability Words		*t*/*F*	*p*
	AB-C	A-BC	AB-C	A-BC		
segmentation accuracy	93.36 (0.13)	93.75 (0.10)	92.58 (0.12)	87.89 (0.13)	1.63	0.19
contextual predictability	5.82 (0.48)		4.17 (0.49)		19.28	<0.001
fluency	5.85 (0.52)	5.78 (0.46)	5.92 (0.53)	5.87 (0.54)	0.41	0.74

Note. Means and standard deviations (SD), where SD are provided in brackets ().

**Table 3:** Examples of Experimental Materials.

	Contextual Predictability	
A-BC	high	春天是个姹紫嫣红的季节，后院的花/生长得很旺盛 (Spring is a season full of vibrant colors, the flowers in the garden grow vigorously)
	low	妈妈喜欢具有观赏价值的植物，后院的花/生长得很旺盛 (Mom likes plants with ornamental value, the flowers in the garden grow vigorously)
AB-C	high	妈妈特别爱吃自家种的果仁，后院的花生/长得很旺盛 (Mom loves eating home-grown nuts, the peanuts in the garden grow vigorously)
	low	妈妈特别会种植农作物，后院的花生/长得很旺盛 (Mom is very good at growing crops, the peanuts in the garden grow vigorously)

## Data Availability

The final data and analysis files can be retrieved from https://osf.io/stcyh/overview?view_only=5c5afca0fd514e98829c61642dbb6edd (accessed on 16 November 2025).
